# An Objectionable Clause

**Published:** 1884-12

**Authors:** W. C. Starbuck


### AN OBJECTIONABLE CLAUSE.

BY W. C. STARBUCK, D. D. S.

At the last meeting of the American Dental Association a suggestive report was presented in reference to some changes in the period of membership. This report, the committee state, "had been prepared hurriedly, and was submitted in a crude form in order to get the views of others."

While approving a portion of the report I most earnestly protest against the adoption of those parts of it where it is recommended that "the printed transactions for each year be sent *only* to members present at that meeting," and that "any permanent member who fails to attend the annual meeting for three consecutive years shall cease to be a member."

The amended constitution up to and including the session of 1882, provides, Article III., Section 5, that permanent members shall consist of those who shall have served *one year* as delegates and complied with the requirements of the Association.

Had I known or suspected that any such measure as the one above quoted was likely to be presented to the Association, I most certainly should not have become a permanent member.

The adoption of a rule that any member who fails to be present —no matter from what cause—for three consecutive years shall be debarred from membership would, no doubt, be a cause for regret to many of the members.

On account of illness I have not been able to attend some of the meetings; but I have promptly paid my annnal dues, and consequently I feel that I am entitled, as long as I am a member in good standing, to the "right and privilege" of having a copy of the printed transactions, and of remaining a member of the Association as long as I comply with its rules and regulations as they now exist. Should the suggestion above referred to, made by the committee, be adopted by the Association, I shall undoubtedly send in my resignation.

